# Unmet palliative care needs and determinants among women with breast and cervical cancer and their caregivers in Ethiopia

**DOI:** 10.3389/fonc.2026.1788481

**Published:** 2026-04-13

**Authors:** Kalkidan Solomon Deribe, Sarona Shewaye, Abigiya Wondimagegnehu, Yoseph Mamo Azmera, Adamu Addissie, Eva J. Kantelhardt, Mirgissa Kaba

**Affiliations:** 1 College of Health Sciences, School of Public Health, Addis Ababa University, Addis Ababa, Ethiopia; 2 Global and Planetary Health Working Group, Institute of Medical Epidemiology, Biostatistics and Informatics, Martin Luther University Halle Wittenberg, Halle (Saale), Germany; 3 Global Health Partnership, Addis Ababa, Ethiopia; 4 Department of Gynaecology, Martin Luther University Halle Wittenberg, Halle (Saale), Germany

**Keywords:** breast cancer, caregivers, cervical cancer, Ethiopia, health disparities, palliative care

## Abstract

**Introduction:**

Breast and cervical cancers are the leading causes of cancer related morbidity and mortality among Ethiopian women. These cancers carry burdens including impacts on femininity, marital relationships, and family stability. Yet palliative care (PC) remains under addressed.

**Objective:**

This study assessed unmet PC needs and determinants among women with breast and cervical cancer and caregiver burden in Ethiopia.

**Methods:**

Institution based cross sectional study was conducted from February to April, 2025 at Tikur Anbessa Specialized Hospital, and Hawassa University Comprehensive Specialized Hospital, Ethiopia. Using systematic random sampling, 480 patients and 458 caregivers were enrolled. Data were collected using Supportive and Palliative Care Indicators Tool for Low Income Settings, African Palliative Care Association tool, and the three item Oslo Social Support Scale. Multivariable logistic regression identified factors associated with higher PC needs. Statistical significance set at p ≤ 0.05.

**Results:**

Using the mean as the threshold for dichotomisation, 261 patients (54.4%) were having higher PC needs. Psychosocial distress was prevalent domain (72.1%), followed by economic hardship (62.3%), physical symptoms (56.7%), and spiritual concerns (52.9%). Breast cancer patients reported higher psychosocial distress than cervical cancer patients (75.6% *vs*. 66.5%, p = 0.03). Patients in Sidama had higher unmet needs (74.6%) than in Addis Ababa (51.5%, p = 0.001). Among cervical cancer patients, chemotherapy receipt was the predictor (AOR = 4.21, 95% CI: 2.18–8.13); among breast cancer patients, late stage diagnosis was associated (AOR = 2.84, 95% CI: 1.52–5.31). Difficulty accessing hospitals (AOR = 1.89, 95% CI: 1.02–3.66), preference for local PC (AOR = 2.65, 95% CI: 1.15–6.10), and Muslim religious affiliation for cervical cancer (AOR = 2.35, 95% CI: 1.02–5.41) and breast cancer (AOR = 2.18, 95% CI: 1.01–4.70) were linked to higher PC needs. Social support was protective (AOR = 0.30). 75.5% caregiver reported high emotional strain and 59.8% high financial burden.

**Conclusion:**

Psychosocial distress was the most common need requiring comprehensive PC service. Given the protective role of social support and patient preference for local care, community based PC models should be integrated into primary health systems. The caregiver burden demands investment in caregiver support programs.

## Introduction

Breast and cervical cancers are the most prevalent malignancies among women worldwide, with over 2 million combined cases and 626, 679 deaths reported in 2022 (1). In Ethiopia, these cancers represent a particularly heavy burden, as they are the leading causes of cancer-related morbidity and mortality among women, yet they remain significantly under-addressed in palliative care (PC) research and service provision. Both cancers are amenable to early detection and curative treatment, yet in Ethiopia, they are typically diagnosed at advanced stages due to limited screening programs and delayed presentation (2). Furthermore, it linked with social and cultural implications, including stigma related to reproductive health, impacts on femininity and marital relationships, and unique symptom burdens that necessitate comprehensive PC approaches (3). Unlike other cancers that may affect different populations or have distinct trajectories, breast and cervical cancers disproportionately affect women during their productive years, creating cascading effects on family stability, child-rearing, and household economies that extend beyond the individual patient to their caregivers (4).

An estimated 56.8 million people requiring PC services annually worldwide (5). However, global disparities in access remain a challenge. Approximately 80% of patients lack adequate pain relief, and only 14% of those in need receive any PC services (6). In low- and middle-income countries where 76% of global PC demand arises the situation is particularly dire, constrained by limited professional training, poor awareness, absence of supportive policies, and fragmented service delivery across all levels of care (7, 8). In Sub-Saharan Africa, only 5% of those in need receive PC, despite the rapidly growing burden of non-communicable diseases including cancer (9).

The specific PC needs of women with breast and cervical cancer in Ethiopia remain understudied, shows a knowledge gap that this research addresses. While existing studies have documented general challenges in PC availability and pain management, they have not systematically characterized the multidimensional unmet needs of patients with these specific cancers or examined the interdependent relationship between patient and caregiver requirements (10).

A 2014 study conducted in Addis Ababa hospitals revealed major gaps, finding that 65% of patients with cancer admitted to a tertiary referral facility received inadequate pain management, with most diagnosed at advanced stages (11). More recently, a rapid program evaluation in Addis Ababa and the Sidama region reported that female patients and their caregivers appreciated available PC services but found them insufficient to address their full range of needs, particularly regarding pain management and psychosocial support (12).

Despite, PC has gained increasing recognition as both a fundamental human right and a public health priority, Ethiopia’s PC services remain fragmented and highly centralized with most specialized care concentrated in Addis Ababa, leaving vast rural populations underserved (13). The country’s diverse ethnic and religious composition distributed across distinct geographic regions, creates varied cultural perceptions of illness, death, and caregiving that shape PC needs and care-seeking behaviours in ways not captured by studies from more homogeneous settings (14). Furthermore, Ethiopia’s health system structure, built around a primary care platform with health extension workers at the community level, offers both challenges and opportunities for PC integration that differ from other African nations with different health system architectures (15).

The caregiver burden in this context needs specific attention because Ethiopian families operate within a collectivist cultural framework where extended family members assume primary responsibility for patient care, often with minimal formal support (16). Caregivers of women with breast and cervical cancer face compounded challenges: they must navigate geographic barriers to access treatment centres, manage the financial strain of prolonged illness, provide physical care for symptoms that may include pain, immobility, and disfigurement, and cope with their own emotional distress while witnessing their loved one’s suffering (17). Importantly, caregiver, and patient needs are intrinsically linked in a bidirectional relationship. Caregiver strain directly affects the quality of care patients receive, influences treatment adherence, and impacts patient psychological well-being, while uncontrolled patient symptoms exacerbate caregiver burden and distress (18).

Studies from Uganda and Kenya have demonstrated that comprehensive PC integrated into oncology services improves symptom control and quality of life (19, 20),. A systematic review of PC in low- and middle-income countries identified the need for context-specific research that accounts for local cultural, economic, and health system factors (21). In Ethiopia specifically, studies have documented that patients face substantial barriers including transportation costs, distance to facilities, and limited service availability, but have not quantified how these factors translate into unmet palliative care needs or examined their variation across urban and rural settings (22, 23).

While it is known that PC services are inadequate, the specific dimensions of need; physical, psychosocial, spiritual, and economic remain poorly characterized, as do the patient-level, caregiver-level, and health system factors that predict higher needs. Furthermore, the ways in which caregiver burden manifests and interacts with patient needs in this cultural and resource-constrained context has not been systematically examined. Therefore, the primary objective of this study was to assess the magnitude of unmet PC needs among women with breast and cervical cancer attending two major Ethiopian referral hospitals. Secondary objectives included: (1) examining regional and cancer type variations in PC needs between urban (Addis Ababa) and semi-urban/rural (Sidama) settings, (2) identifying independent sociodemographic, clinical, psychosocial, and health system factors associated with higher PC needs, and (3) describing the magnitude, and nature of caregiver burden to inform decentralized service planning.

This study is conceptually framed by the World Health Organization’s (WHO) PC domains which recognize that comprehensive PC must address physical symptoms, psychological distress, social support, and spiritual concerns as interconnected dimensions of suffering (24).

## Methods

### Study design

An institution-based cross-sectional study was conducted. The reporting of this study follows the Strengthening the Reporting of Observational Studies in Epidemiology (STROBE_v4) guidelines for cross-sectional studies to ensure comprehensive and transparent reporting (Annex_1).

### Study area and time

The study was conducted from February to April 2025 at Tikur Anbessa Specialized Hospital (TASH) in Addis Ababa and Hawassa University Comprehensive Specialized Hospital (HUCSH) in the Sidama region. TASH is a major national cancer referral centre with a high patient load, a diverse patient population, and a wide range of oncology services, making it a suitable site for this research. HUCSH is a federally funded cancer centre serving more than 20 million people in Ethiopia, providing care to a broad patient population Both hospitals provide specialized oncology services, including chemotherapy, radiation therapy, brachytherapy, and PC.

These two hospitals serve geographically distinct catchment areas within Ethiopia’s hierarchical public health referral system. Patients attending HUCSH are from Sidama and surrounding southern regions, while those attending TASH are from Addis Ababa and adjacent areas due to the structured referral pathway where patients are referred from primary health centres to district hospitals and then to their nearest regional referral hospital. This geographic specificity justifies the use of these institutions as proxies for regional comparisons in this study.

### Study population

The study population included all women with confirmed breast or cervical cancer attending TASH and HUCSH, as well as their primary caregivers, during the data collection period.

#### Eligibility criteria

Women aged ≥18 years with a clinical or pathological diagnosis of breast or cervical cancer at any stage of illness, including those with terminal or late-stage disease, attending outpatient or inpatient care, who were able to provide informed consent and communicate adequately, were eligible for inclusion. A clinical diagnosis was defined as a diagnosis made by a specialist physician (oncologist or gynaecologist) based on clinical examination and imaging findings, while a pathological diagnosis was confirmed through histopathological or cytological examination. Women who were critically ill, had severe psychiatric disorders, were attending their first diagnostic visit without a confirmed diagnosis, or were in the immediate post-procedural period were excluded.

The primary caregiver was defined as the individual (family member or other) who provided the majority of unpaid, daily care and support to the patient, including assistance with activities of daily living, medical care, emotional support, or financial management, and was identified by the patient as their main caregiver. Caregivers who were not directly or fully involved in providing care, were excluded. When a patient did not have an available primary caregiver, the patient was retained in the patient analysis, and caregiver data were coded as missing, this approach was taken to avoid excluding patients based on caregiver availability and to reflect the reality that some patients lack identifiable caregivers. Family caregivers who were cancer survivors were not excluded from participation. However, their survivor status was recorded as part of the caregiver health status variable to allow for sensitivity analyses examining whether survivor status influenced reported caregiver burden or patient needs.

### Sample size determination and sampling procedure

For the primary objective of estimating the proportion of patients with higher PC needs, the minimum required sample was calculated using the single population proportion formula with the following assumptions: Z = 1.96 (95% confidence interval), p = 0.5 (conservative estimate), and margin of error of 5%. The initial sample size without finite population correction was: n⁰ = (Z² × p(1-p))/d² = (1.96² × 0.5 × 0.5)/(0.05)² = 384 patients.

A finite population correction was applied because the total population of women with breast and cervical cancer across both facilities was 1, 312 (less than 10, 000), comprising 982 women with breast cancer (792 at TASH, 190 at HUCSH) and 330 women with cervical cancer (298 at TASH, 32 at HUCSH). The correction formula used was: n = n⁰/[1 + ((n⁰ - 1)/N)], where n⁰ = 384 is the initial sample size without correction and N = 1, 312 represents the total population size. This yielded n = 384/[1 + ((384 - 1)/1, 312)] = 384/[1 + (383/1, 312)] = 384/1.292 = 297 patients. Adding 10% for non-response gave a minimum required sample of 327 patients for the primary objective.

To address secondary objectives requiring subgroup analyses, the sample was increased with specific targets. Cervical cancer patients constitute only 25% of the population (330/1, 312) but require separate analysis due to distinct clinical and psychosocial characteristics. To enable cancer type-specific multivariable regression with 10–15 predictors, a minimum of 100 events (patients with higher PC needs) was required. Assuming approximately 50% of cervical cancer patients would have higher PC needs, we needed approximately 185 cervical cancer patients (56% sampling fraction). For breast cancer patients, to maintain a 1:1 patient-caregiver ratio for dyad analysis and enable cancer-specific multivariable modelling with 15–20 predictors, we targeted 295 patients (30% sampling fraction from the breast cancer population of 982). To detect anticipated regional differences between Sidama and Addis Ababa (expected 75% *vs*. 52% higher PC needs based on pilot data) with 80% power, we needed minimum 50 patients from Sidama and recruited 59.

The final sample of 480 patients (295 breast cancer, 185 cervical cancer) exceeds the minimum required for the primary objective (327) and provides adequate power for all secondary objectives: 94% power to detect regional differences, events-per-variable ratios of 10-13.4 for cancer-specific models, and 85% power for caregiver burden comparisons. The overall sampling fraction for cervical cancer (185/330 = 0.56) was higher than for breast cancer (295/982 = 0.30) to ensure adequate representation of cervical cancer patients for meaningful subgroup analyses.

The sample was proportionally allocated to each health institution using institute-specific population proportions. From HUCSH, 47 women with breast cancer and 12 women with cervical cancer were recruited, along with their caregivers. From TASH, 248 women with breast cancer and 173 women with cervical cancer were included, along with their caregivers. A one-to-one patient-caregiver ratio was applied. When a woman did not have an available primary caregiver, she was retained in the patient analysis and caregiver data were coded as not available.

### Sampling technique and procedure

Participants were selected using systematic random sampling based on medical record numbers from the oncology clinic registries, which served as the sampling frame. The sampling frame was compiled by reviewing all medical records of women with breast and cervical cancer who attended the oncology clinics at both hospitals during the six-month period prior to data collection (August 2024 to January 2025). This period was selected to ensure a complete and up-to-date list of eligible patients. The sampling interval for each cancer type at each facility was calculated by dividing the total number of eligible women by the required sample size for that facility. This resulted facility-specific sampling intervals: at TASH, every 3rd breast cancer patient (interval 3.19 rounded to 3) and every 2nd cervical cancer patient (interval 1.72 rounded to 2); at HUCSH, every 4th breast cancer patient (interval 4.04 rounded to 4) and every 3rd cervical cancer patient (interval 2.67 rounded to 3). The first participant at each facility and for each cancer type was selected using a lottery method to ensure randomness, with subsequent selections made by adding the sampling interval to the previous selection.

### Study variables

The primary outcome variable was unmet palliative care (PC) need, which was dichotomised using the mean SPICT-LIS score to classify patients as having higher PC needs (scores ≥ mean) versus lower PC needs (scores < mean). Independent variables included socio-demographic factors (age, marital status, education, employment, income, religion), clinical variables (cancer type, stage, treatment modality), psychosocial variables, cultural factors (use of traditional medicine), environmental factors (place of residence, region), and health system-related variables (difficulty accessing hospital, distance to hospital, transportation affordability, availability of PC services, medical help-seeking behaviour, receipt of pain relief, preferred location for PC). Social support was measured using the three-item Oslo Social Support Scale (OSSS-3), which assesses number of close confidants, sense of concern from others, and availability of practical help from neighbours. Total scores range from 3 to 14, with respondents categorised as having poor social support (scores 3-8), moderate social support (scores 9-11), or strong social support (scores 12-14) based on established cutoffs. Caregiver burden was described in terms of caregiving demands, emotional strain, and financial burden, assessed using the APCA Caregiver Burden Module.

### Data collection tools, and measurements

The Supportive and Palliative Care Indicators Tool for Low-Income Settings (SPICT-LIS) was used to assess unmet PC needs across 22 items covering physical symptoms, psychosocial distress, spiritual concerns, and economic hardship. Total scores were dichotomised using the sample mean to classify higher versus lower PC needs (Annex_2).

The 10 item African Palliative Care Association (APCA) Palliative Outcome Scale was used to assess PC outcomes and caregiver burden. The APCA Caregiver Burden Module added items assessing emotional strain, financial burden, and caregiving demands, rated on a 5-point Likert scale with higher scores indicating greater burden (Annex_2).

The three-item Oslo Social Support Scale (OSSS-3) measured perceived social support regarding number of confidants, concern from others, and availability of practical help. Total scores of 3–8, 9–11, and 12–14 indicated poor, moderate, and strong social support, respectively. Additional questionnaire items assessed sociodemographic, clinical, health system, and cultural factors, developed through literature review (Annex_2).

All tools were translated into Amharic and Sidama using forward-backward translation, with content validity confirmed by three Ethiopian palliative care experts and face validity established through pretesting with 48 patients and their caregivers at St. Paul’s Millennium Medical College. The SPICT-LIS showed good reliability (Cronbach’s α = 0.84), the APCA Palliative Outcome Scale showed acceptable reliability for both patient and caregiver versions (Cronbach’s α = 0.81 and 0.79, respectively), and the OSSS-3 also demonstrated acceptable reliability (Cronbach’s α = 0.76).

### Data collection procedure

Data were collected using a pretested, interviewer-administered structured questionnaire comprising multiple validated instruments. The questionnaires were initially prepared in English and subsequently translated into Amharic and Sidama by qualified language experts. To ensure accuracy and consistency, the tools were back-translated into English by independent translators.

Data collection was conducted through phone interviews. Prior to the interviews, informed consent was obtained by oncology nurses working in the oncology units of the two study facilities. These nurses also collected participants’ phone numbers from the hospital health management information systems. The data collection process was closely supervised by the principal investigator. Participants were selected from the patient registry books, and interviews were conducted by six experienced BSc-level nurse data collectors.

### Data management and analysis

Data were exported from ODK to Stata version 17 for analysis. Descriptive statistics were used to summarise frequency distributions, proportions, and measures of central tendency (mean, median) and dispersion (standard deviation, interquartile range) for socio-demographic characteristics, clinical variables, and caregiver burden indicators.

For the primary outcome of palliative care need, total scores on the SPICT-LIS were calculated for each patient. Using the mean score as the threshold for dichotomisation, patients with scores equal to or greater than the mean were classified as having higher PC needs, while those with scores below the mean were classified as having lower PC needs. This dichotomised variable higher PC needs (yes/no) served as the primary outcome for all bivariate and multivariable analyses.

Bivariate analyses were conducted to examine associations between independent variables and the primary outcome. Chi-square tests were used for categorical variables (including cancer type, region, cancer stage, treatment modality, religion, difficulty accessing hospital, medical help-seeking behaviour, receipt of pain relief, preferred location for PC, and social support level). For caregiver burden analysis, chi-square tests were used to compare proportions of high emotional strain and high financial burden between regions.

Binary logistic regression was used to generate crude odds ratios (COR) and adjusted odds ratios (AOR) with 95% confidence intervals (CI) as measures of association for higher PC needs. Variables with a p-value of ≤0.2 in the bivariate analysis were included in the multivariable model, along with variables of theoretical importance based on literature review. Multivariable logistic regression was performed separately for cervical cancer and breast cancer patients to identify cancer type-specific determinants, with higher PC needs as the outcome variable in both models, as preliminary analysis suggested differential patterns of association.

Model building employed a stepwise forward selection approach, with variables retained in the final model if they demonstrated statistical significance at p ≤ 0.05 or substantially improved model fit. The Hosmer-Lemeshow goodness-of-fit test was used to assess model adequacy, with p > 0.05 indicating good fit. Multicollinearity was assessed using variance inflation factors (VIF), with values >10 indicating problematic collinearity. Interaction terms between cancer type and key predictors (chemotherapy, stage, region) were tested to examine whether associations differed significantly between breast and cervical cancer patients.

For caregiver burden analysis, emotional strain and financial burden scores from the APCA Caregiver Burden Module were dichotomised using the threshold of ≥3 (on a 0–4 scale) to indicate high burden, based on established cutoffs in the literature. Regional comparisons of caregiver burden proportions were conducted using chi-square tests, and odds ratios with 95% CI were calculated to quantify the magnitude of regional differences. Statistical significance was set at p ≤ 0.05 for all analyses.

### Data quality assurance

The questionnaire was pretested on 10% of the sample at Saint Paul’s Millennium Medical College 1 month prior to data collection to assess item clarity. Modifications were made based on the pretest findings. Oncology nurses from each health facility were assigned to obtain informed consent. A 2-day training on the study objectives, methodology, data collection tools, and procedures was provided by the principal investigator to the six data collectors. Continuous supervision was conducted throughout the data collection period. Data were checked daily for completeness by both the data collectors and the principal investigator. Intensive data cleaning was performed prior to analysis.

## Results

A total of 480 patients and 458 caregivers (95.4% response rate) completed the interviews and were included in the final analysis.

### Socio-demographic characteristics of patients

The patients’ mean age was 46.6 years (standard deviation [SD] = 11.4), with ages ranging from 18 to 82 years. The majority of patients were married (n = 296, 61.8%), and most had completed primary education (n = 147, 30.6%). Employment status varied, with self-employment (n = 119, 24.8%) and daily labour (n = 122, 25.5%) being the most common occupations. The mean monthly household income was 4, 254.7 Ethiopian birr (ETB) (SD = 5, 821.8), and the average household size was five members (SD = 2.5). A large proportion of patients were enrolled in community-based health insurance (n = 347, 72.2%) and resided in urban areas (n = 350, 72.8%). More than half of the respondents identified as Orthodox Christian (n = 265, 55.2%) (**Table 1**).

**Table 1:** Socio-demographic characteristics of women with breast and cervical cancer in Ethiopia, 2025.

Characteristic	Category	Frequency (n=480)	Percentage (%)
Age (years)	Mean ± SD	46.6 ± 11.4	
	Range	18–82	
Marital status	Married	296	61.8
	Widowed	82	16.9
	Divorced	60	12.5
	Single	42	8.8
Educational status	Primary education	147	30.6
	Illiterate	131	27.4
	Higher than secondary	111	23.1
	Secondary education	91	18.9
Employment status	Unemployed	132	27.5
	Daily labour	122	25.5
	Self-employed	119	24.8
	Employed	107	22.2
Monthly household income (ETB)	Mean ± SD	4, 254.7 ± 5, 821.8	
Household size (persons)	Mean ± SD	5.0 ± 2.5	
Community-based health insurance	Yes	347	72.2
	No	133	27.8
Place of residence	Urban	350	72.8
	Rural	130	27.2
Religion	Orthodox Christian	265	55.2
	Protestant Christian	109	22.7
	Muslim	97	20.2
	Catholic	9	1.9

ETB, Ethiopian birr; SD, Standard deviation.

### Socio-demographic characteristics of caregivers

Of the 480 patients enrolled, 458 (95.4%) had an identified primary caregiver who participated in the study, while 22 patients (4.6%) had no available primary caregiver. The mean age of caregivers was 38.0 years (SD = 12.6), ranging from 21 to 74 years. Slightly more than half of the caregivers were female (n = 248, 54.1%). The majority of caregivers were married (n = 282, 61.6%). Nearly half had attained education above secondary level (n = 213, 46.5%), while 49 caregivers (10.7%) were unable to read or write. Approximately one-third of caregivers were parents (n = 136, 29.7%), followed by spouses (n = 100, 21.9%) (**Table 2**).

**Table 2:** Socio-demographic characteristics of caregivers of women with breast and cervical cancer in Ethiopia, 2025.

Characteristic	Category	Frequency (n=458)	Percentage (%)
Caregiver age (years)	Mean ± SD	38.0 ± 12.6	
	Range	21–74	
Caregiver sex	Female	248	54.1
	Male	210	45.9
Family size (persons)	Mean ± SD	5.0 ± 1.9	
Children aged <15 years (n)^a^	Mean ± SD	2.0 ± 1.6	
Marital status	Married	282	61.6
	Single	146	31.9
	Widowed	14	3.0
	Separated or divorced	16	3.5
Educational status	Higher than secondary	213	46.5
	Secondary school	125	27.3
	Primary school	71	15.5
	Illiterate	49	10.7
Occupation	Employed	165	36.0
	Daily labourer	140	30.7
	Work at own home	66	14.4
	Unemployed	62	13.5
	Farmer	25	5.4
Religion	Orthodox Christian	240	52.4
	Protestant Christian	120	26.2
	Muslim	92	20.1
	Catholic	6	1.3
Relationship with patient	Parent	136	29.7
	Spouse	100	21.9
	Sibling	99	21.6
	Child	76	16.6
	Friend	47	10.2

SD, Standard deviation; ^a^ This variable represents the number of dependent children under 15 years living in the caregiver’s household.

### Socio-cultural, health system, and access barrier factors

Use of traditional medicine was reported by 312 patients (65.0%) overall, with higher rate in Sidama (n = 44, 75.0%) compared with Addis Ababa (n = 268, 63.6%) (p = 0.082). Mean monthly household income was substantially lower in Sidama (3, 800 ETB) compared with Addis Ababa (5, 200 ETB).

Service accessibility was reported as inadequate by 332 patients (69.2%), with similar proportions in both regions (Sidama: n = 42, 71.2%; Addis Ababa: n = 290, 68.9%; p = 0.719). Availability of PC services was reported by 253 patients (52.7%) overall, with higher availability in Addis Ababa (n = 229, 54.5%) compared with Sidama (n = 24, 41.7%) (p = 0.058). Care-related delays were experienced by 244 patients (50.8%), and long waiting times were reported by 352 patients (73.3%). Transportation availability was lacking for 343 patients (71.5%), and cost-related barriers to care were reported by 229 patients (47.7%).

Difficulty accessing the hospital was reported by 254 patients (52.9%), with significantly higher proportions in Sidama (n = 44, 75.0%) compared with Addis Ababa (n = 210, 49.9%) (p = 0.001). Long distance to the hospital was reported by 259 patients (54.0%), affecting 44 patients (75.0%) in Sidama versus 215 patients (51.1%) in Addis Ababa (p = 0.001).

Of 480 patients, 299(62.3%) reporting inability to afford transport costs, 44 patients (75.0%) in Sidama and 255 patients (60.6%) in Addis Ababa (p = 0.032). Physical access barriers (terrain, road conditions, lack of vehicles) were reported by 120 patients (25.0%), with higher rates in Sidama (n = 20, 33.9%) than in Addis Ababa (n = 100, 23.8%) (p = 0.087). (**Table 3**).

**Table 3:** Socio-cultural, health system, and access barriers factors among women with breast and cervical cancer by region, Ethiopia, 2025.

Factor	Category	Sidama (n = 59)	Addis Ababa (n=421)	Total (N = 480)	P-value
		n (%)	n (%)	n (%)	
Difficulty accessing hospital	Yes	44 (75.0)	210 (49.9)	254 (52.9)	0.001*
	No	15 (25.0)	211 (50.1)	226 (47.1)	
Long distance to hospital	Yes	44 (75.0)	215 (51.1)	259 (54.0)	0.001*
	No	15 (25.0)	206 (48.9)	221 (46.0)	
Transportation affordability	Unaffordable	44 (75.0)	255 (60.6)	299 (62.3)	0.032*
	Affordable	15 (25.0)	166 (39.4)	181 (37.7)	
Physical access barriers	Yes	20 (33.9)	100 (23.8)	120 (25.0)	0.087
	No	39 (66.1)	321 (76.2)	360 (75.0)	
Service accessibility	Inadequate	42 (71.2)	290 (68.9)	332 (69.2)	0.719
	Adequate	17 (28.8)	131 (31.1)	148 (30.8)	
Availability of PC services	Available	24 (41.7)	229 (54.5)	253 (52.7)	0.058
	Not available	35 (58.3)	192 (45.5)	227 (47.3)	
Transportation availability	Not available	45 (76.3)	298 (70.8)	343 (71.5)	0.373
	Available	14 (23.7)	123 (29.2)	137 (28.5)	
Cost-related barriers to care	Yes	33 (55.9)	196 (46.6)	229 (47.7)	0.173
	No	26 (44.1)	225 (53.4)	251 (52.3)	
Care-related delays	Yes	31 (52.5)	213 (50.6)	244 (50.8)	0.778
	No	28 (47.5)	208 (49.4)	236 (49.2)	
Long waiting times	Yes	44 (74.6)	308 (73.2)	352 (73.3)	0.816
	No	15 (25.4)	113 (26.8)	128 (26.7)	
Physical infrastructure support	Inadequate	29 (49.2)	216 (51.3)	245 (51.0)	0.755
	Adequate	30 (50.8)	205 (48.7)	235 (49.0)	
Use of traditional medicine	Yes	44 (75.0)	268 (63.6)	312 (65.0)	0.082
	No	15 (25.0)	153 (36.4)	168 (35.0)	
Monthly household income (ETB)	Mean ± SD	3, 800	5, 200	4, 254.7 ± 5, 821.8	–

PC, palliative care; ETB, Ethiopian birr; Significant difference between regions at p < 0.05 (chi-square test)*, all variables are based on patient self-report of perceived experiences.

### Palliative care needs, distribution of social support, and regional variations among women with breast and cervical cancer

Based on the composite PC needs score from the SPICT-LIS, the mean score was 11.4 (SD = 4.8, range: 3–21). Using the mean as the threshold for dichotomisation, 261 patients (54.4%, 95% CI: 49.8%–58.9%) were categorized as having higher PC needs (scores ≥11.4), while 219 patients (45.6%, 95% CI: 41.1%–50.2%) were categorised as having lower PC needs.

Of the 480 patients, 295 (61.5%) had breast cancer and 185 (38.5%) had cervical cancer. When stratified by cancer type, the magnitude of higher PC needs was similar between the two groups. Among breast cancer patients (n = 295), 161 patients (54.6%, 95% CI: 48.8%–60.3%) were classified as having higher PC needs. Among cervical cancer patients (n = 185), 100 patients (54.1%, 95% CI: 46.6%–61.4%) were classified as having higher PC needs. This difference was not statistically significant (χ² = 0.01, p = 0.91).

Unmet PC needs were significantly higher in Sidama (44 of 59 patients, 74.6%) than in Addis Ababa (217 of 421 patients, 51.5%) (χ² = 11.2, p = 0.001). Among breast cancer patients, those in Sidama had significantly higher PC needs (35 of 47 patients, 74.5%) compared with those in Addis Ababa (126 of 248 patients, 50.8%) (χ² = 8.9, p = 0.003). Among cervical cancer patients, those in Sidama also showed higher needs (9 of 12 patients, 75.0%) compared with those in Addis Ababa (91 of 173 patients, 52.6%), though this difference did not reach statistical significance (χ² = 2.3, p = 0.13), likely due to the small sample size of cervical cancer patients in Sidama.

Regarding social support measured by the Oslo Social Support Scale, the mean score was 8.7 (SD = 2.9, range: 3–14). Based on established cutoffs, 224 respondents (46.7%) reported poor social support (scores 3–8), 182 (38.0%) reported moderate support (scores 9–11), and 74 (15.3%) reported strong social support (scores 12–14).

When examined by cancer type, the distribution of social support was similar. Among breast cancer patients, 137 (46.4%) reported poor support, 112 (38.0%) reported moderate support, and 46 (15.6%) reported strong support. Among cervical cancer patients, 87 (47.0%) reported poor support, 70 (37.9%) reported moderate support, and 28 (15.1%) reported strong support, with no significant difference between cancer types (χ² = 0.03, p = 0.98).

Regional variations in social support were observed, with Sidama patients reporting poorer support overall. In Sidama, 31 patients (52.5%) had poor support, 20 (33.9%) had moderate support, and 8 (13.6%) had strong support, compared with Addis Ababa where 193 patients (45.8%) had poor support, 162 (38.5%) had moderate support, and 66 (15.7%) had strong support, though this difference was not statistically significant (χ² = 1.1, p = 0.58).

The protective effect of social support against higher PC needs was evident across both cancer types. Among breast cancer patients with strong social support, only 14 of 46 (30.4%) had higher PC needs, compared with 89 of 137 (65.0%) with poor support. Similarly, among cervical cancer patients with strong social support, only 10 of 28 (35.7%) had higher PC needs, compared with 59 of 87 (67.8%) with poor support. (**Table 4**).

**Table 4:** Palliative care needs, social support, and regional variations among women with breast and cervical cancer in Ethiopia, 2025.

Characteristic	Category	Sidama (n = 59)	Addis Ababa (n = 421)	Total (N = 480)	P-value
		n (%)	n (%)	N (%)	
PC needs score (mean ± SD)				11.4 ± 4.8	
Higher PC needs (overall)	Yes	44 (74.6)	217 (51.5)	261 (54.4)	0.001*
	No	15 (25.4)	204 (48.5)	219 (45.6)	
Higher PC needs by cancer type^a^					
	BC - Yes	35/47 (74.5)	126/248 (50.8)	161/295 (54.6)	0.003*
	CC - Yes	9/12 (75.0)	91/173 (52.6)	100/185 (54.1)	0.13
Social support level	Poor (scores 3–8)	31 (52.5)	193 (45.8)	224 (46.7)	0.58
	Moderate (scores 9–11)	20 (33.9)	162 (38.5)	182 (38.0)	
	Strong (scores 12–14)	8 (13.6)	66 (15.7)	74 (15.3)	
Social support by cancer type					
	BC- Poor support			137/295 (46.4)	0.98
	BC - Moderate support			112/295 (38.0)	
	BC - Strong support			46/295 (15.6)	
	CC- Poor support			87/185 (47.0)	
	CC - Moderate support			70/185 (37.9)	
	CC- Strong support			28/185 (15.1)	
Protective effect of social support					
	BC - Higher PC needs with strong support			14/46 (30.4)	
	BC - Higher PC needs with poor support			89/137 (65.0)	
	CC - Higher PC needs with strong support			10/28 (35.7)	
	CC - Higher PC needs with poor support			59/87 (67.8)	

PC, palliative care; SD, standard deviation; Significant difference between regions at p < 0.05 (chi-square test)*, a Data for higher PC needs by cancer type are presented as n/N (%), where the denominator represents the total number of patients with that cancer type in each region.

### Palliative care needs by stage, treatment, and region among women with breast and cervical cancer

Late-stage diagnosis (stages III–IV) was reported in 213 patients (44.4%), with a higher proportion among cervical cancer patients (93 of 185, 50.3%) compared with breast cancer patients (120 of 295, 40.7%). Chemotherapy was the most common treatment modality, received by 335 patients (69.8%), including 123 cervical cancer patients (66.5%) and 212 breast cancer patients (71.9%).

Regional distribution showed that 421 patients (87.7%) were recruited from Addis Ababa, and 59 patients (12.3%) from Sidama region. Patients in Sidama had a higher proportion of late-stage diagnosis (34 of 59, 57.6%) compared with those in Addis Ababa (179 of 421, 42.5%).

Analysis of clinical characteristics revealed that both cancer stage and treatment modality were significantly associated with the level of PC needs across both cancer types. Among patients receiving chemotherapy (n = 335), 215 (64.2%) had higher PC needs, compared with 46 of 145 patients (31.7%) not receiving chemotherapy (χ² = 42.3, p < 0.001). Late-stage diagnosis was associated with higher PC needs, with 143 of 213 late-stage patients (67.1%) having higher needs compared with 118 of 267 early-stage patients (44.2%) (χ² = 25.1, p < 0.001).

When stratified by cancer type, these patterns remained consistent but with notable differences in magnitude. Among breast cancer patients, those receiving chemotherapy had significantly higher PC needs (123 of 212, 58.0%) compared with those not receiving chemotherapy (38 of 83, 45.8%) (χ² = 4.2, p = 0.04). However, among cervical cancer patients, the association was markedly stronger: chemotherapy recipients reported dramatically higher needs (92 of 123, 74.8%) compared with non-recipients (8 of 62, 12.9%) (χ² = 63.4, p < 0.001).

For cancer stage, late-stage breast cancer patients had higher PC needs (85 of 120, 70.8%) compared with early-stage patients (76 of 175, 43.4%) (χ² = 21.5, p < 0.001). Among cervical cancer patients, those with late-stage disease also reported higher needs (58 of 93, 62.4%) compared with early-stage patients (42 of 92, 45.7%) (χ² = 5.2, p = 0.02).

The association between chemotherapy and higher PC needs persisted after adjusting for cancer stage and other covariates. The direction and magnitude of associations for the significant factors identified in the main analysis were similar across cancer types, with no evidence of interaction by cancer type for any variable (all interaction p-values >0.10) (**Table 5**).

**Table 5:** Palliative care needs by cancer type, stage, treatment, and region among women with breast and cervical cancer in Ethiopia, 2025.

Clinical characteristic	Category	Breast cancer (n = 295)		Cervical cancer (n = 185)		Total (N = 480)	
		n/N	% with higher needs	n/N	% with higher needs	N	% with higher needs
Overall		161/295	54.6	100/185	54.1	261/480	54.4
Cancer Stage	Early (I–II)	76/175	43.4	42/92	45.7	118/267	44.2
	Late (III–IV)	85/120	70.8	58/93	62.4	143/213	67.1
	p-value		<0.001		0.02*		<0.001
Receiving Chemotherapy	Yes	123/212	58.0	92/123	74.8	215/335	64.2
	No	38/83	45.8	8/62	12.9	46/145	31.7
	p-value		0.04*		<0.001		<0.001
Late-stage diagnosis by region	Sidama	21/47 (44.7%)		9/12 (75.0%)		30/59 (50.8%)	
	Addis Ababa	99/248 (39.9%)		80/173 (46.2%)		179/421 (42.5%)	
	P-value		0.48		0.42		0.027*

Significant difference between stage or treatment categories at p < 0.05 (chi-square test)*.

### Multidimensional assessment of palliative care needs by domain, cancer type, and region

Palliative care needs varied across the four domains of the SPICT-LIS, with distinct patterns observed by cancer type and geographic region. Overall, psychosocial distress was the most frequently reported domain, affecting 346 patients (72.1%), followed by economic hardship (299 patients, 62.3%), physical symptoms (272 patients, 56.7%), and spiritual concerns (254 patients, 52.9%).

Regarding assessing the magnitude of palliative care needs, item-level analysis revealed that, among breast cancer patients (n = 295), the most commonly reported unmet needs were worry about family (243 patients, 82.4%), sadness or depression (232 patients, 78.6%), and changes in appearance (210 patients, 71.2%). Among cervical cancer patients (n = 185), the most frequently reported unmet needs were pain (141 patients, 76.2%), worry about treatment side effects (136 patients, 73.5%), and shame or stigma related to the diagnosis (126 patients, 68.1%). Overall, only 28 patients (5.8%) reported no unmet needs across all domains.

Among breast cancer patients (n = 295), psychosocial distress was reported by 223 patients (75.6%), which was significantly higher than among cervical cancer patients (123 patients, 66.5%) (p = 0.03). For cervical cancer patients (n = 185), physical symptoms were reported by 107 patients (57.8%), compared with 165 breast cancer patients (55.9%), though this difference was not statistically significant (p = 0.68). Spiritual concerns were reported by 102 cervical cancer patients (55.1%) versus 152 breast cancer patients (51.5%) (p = 0.44), while economic hardship affected 118 cervical cancer patients (63.8%) and 181 breast cancer patients (61.4%) (p = 0.60), with no significant differences between cancer types for these domains.

Overall, psychosocial distress was significantly higher in Sidama (49 patients, 83.1%) than in Addis Ababa (297 patients, 70.5%) (p = 0.04). Economic hardship also showed significant regional variation, affecting 44 Sidama patients (74.6%) compared with 255 Addis Ababa patients (60.6%) (p = 0.03).

Physical symptoms were reported by 38 Sidama patients (64.4%) versus 234 Addis Ababa patients (55.6%) (p = 0.20), and spiritual concerns affected 37 Sidama patients (62.7%) compared with 217 Addis Ababa patients (51.5%) (p = 0.10), though these differences did not reach statistical significance.

Breast cancer patients in Sidama reported significantly higher psychosocial distress (41 patients, 87.2%) compared with those in Addis Ababa (182 patients, 73.4%) (p = 0.04). Similarly, economic hardship among breast cancer patients was significantly higher in Sidama (35 patients, 74.5%) than in Addis Ababa (146 patients, 58.9%) (p = 0.04).

Physical symptoms affected 8 cervical cancer patients in Sidama (66.7%) versus 99 in Addis Ababa (57.2%) (p = 0.52); psychosocial distress affected 8 Sidama patients (66.7%) versus 115 Addis Ababa patients (66.5%) (p = 0.99); spiritual concerns affected 8 Sidama patients (66.7%) versus 94 Addis Ababa patients (54.3%) (p = 0.41); and economic hardship affected 9 Sidama patients (75.0%) versus 109 Addis Ababa patients (63.0%) (p = 0.41). None of these cervical cancer-specific regional differences reached statistical significance. (**Table 6**).

**Table 6:** Multidimensional assessment of palliative care needs by domain, cancer type, and region among women with breast and cervical cancer in Ethiopia, 2025.

Palliative care domain	Cancer type	Sidama (n = 59)	Addis Ababa (n = 421)	Total (N = 480)	P-value (region)*	P-value (cancer type)*
		n (%)	n (%)	N (%)		
Physical symptoms	Breast Cancer	30/47 (63.8)	135/248 (54.4)	165/295 (55.9)	0.23	0.68
	Cervical Cancer	8/12 (66.7)	99/173 (57.2)	107/185 (57.8)	0.52	
	Total	38/59 (64.4)	234/421 (55.6)	272/480 (56.7)	0.20	
Psychosocial distress	Breast Cancer	41/47 (87.2)	182/248 (73.4)	223/295 (75.6)	0.04	0.03
	Cervical Cancer	8/12 (66.7)	115/173 (66.5)	123/185 (66.5)	0.99	
	Total	49/59 (83.1)	297/421 (70.5)	346/480 (72.1)	0.04	
Spiritual concerns	Breast Cancer	29/47 (61.7)	123/248 (49.6)	152/295 (51.5)	0.13	0.44
	Cervical Cancer	8/12 (66.7)	94/173 (54.3)	102/185 (55.1)	0.41	
	Total	37/59 (62.7)	217/421 (51.5)	254/480 (52.9)	0.10	
Economic hardship	Breast Cancer	35/47 (74.5)	146/248 (58.9)	181/295 (61.4)	0.04	0.60
	Cervical Cancer	9/12 (75.0)	109/173 (63.0)	118/185 (63.8)	0.41	
	Total	44/59 (74.6)	255/421 (60.6)	299/480 (62.3)	0.03	

p-value from chi-square test; statistical significance set at p < 0.05^*^.

### Determinants for higher palliative care needs by cancer type

Bivariate and multivariable logistic regression analyses were conducted separately for cervical cancer and breast cancer patients to identify factors independently associated with higher PC needs within each cancer type. The final multivariable models included variables with p ≤ 0.2 in bivariate analysis, or retained as confounders based on theoretical importance.

### Determinants among cervical cancer patients

Among cervical cancer patients (n = 185), 100 (54.1%) were classified as having higher PC needs. Women with cervical cancer who identified as Muslim were more than twice as likely to report high PC needs compared with Orthodox Christians (AOR = 2.35, 95% CI: 1.02–5.41, p = 0.04). Difficulty accessing the hospital was associated with twofold higher odds of unmet needs (AOR = 2.01, 95% CI: 1.01–4.00, p = 0.04). Cervical cancer patients who did not seek medical help had more than twice the odds of higher PC needs (AOR = 2.18, 95% CI: 1.01–4.70, p = 0.04). Receipt of pain relief services was also associated with higher needs (AOR = 1.92, 95% CI: 1.01–3.65, p = 0.04).

Preference for receiving PC at a nearby health centre was associated with significantly higher PC needs compared with preference for home-based care (AOR = 2.71, 95% CI: 1.12–6.56, p = 0.02). Strong social support demonstrated a strong protective effect, with cervical cancer patients reporting strong support being 73% less likely to have higher PC needs compared with those reporting poor support (AOR = 0.27, 95% CI: 0.10–0.73, p = 0.01).

Late-stage diagnosis (stages III–IV) was associated with nearly twofold higher odds of unmet PC needs (AOR = 1.89, 95% CI: 1.01–3.54, p = 0.04). Cervical cancer patients receiving chemotherapy were more than four times as likely to report higher PC needs compared with those not receiving chemotherapy (AOR = 4.21, 95% CI: 2.18–8.13, p < 0.001) (**Table 7**).

**Table 7:** Bivariable and multivariable logistic regression analysis of determinants for higher palliative care need among women with breast and cervical cancer in Ethiopia, 2025.

Variable	Category	Cervical Cancer (n = 185)				Breast Cancer (n = 295)			
		Higher PC needs (n = 100)	COR (95% CI)	AOR (95% CI)	P-value	Higher PC needs (n = 161)	COR (95%CI)	AOR (95% CI)	P-value
		n (%)				n (%)			
Religion	Orthodox	51 (51.0)	1.00	1.00		85 (52.8)	1.00	1.00	
	Muslim	29 (29.0)	2.71 (1.28–5.74)	2.35 (1.02–5.41)	0.04*	42 (26.1)	2.52 (1.32–4.81)	2.18 (1.01–4.70)	0.04*
	Protestant	18 (18.0)	0.81 (0.41–1.60)	0.75 (0.34–1.65)	0.48	31 (19.3)	0.74 (0.42–1.31)	0.68 (0.34–1.36)	0.27
	Catholic	2 (2.0)	1.24 (0.20–7.69)	1.12 (0.15–8.36)	0.91	3 (1.9)	1.15 (0.22–6.01)	1.01 (0.16–6.38)	0.99
Difficulty accessing hospital	Yes	68 (68.0)	3.21 (1.82–5.66)	2.01 (1.01–4.00)	0.04*	101 (62.7)	2.89 (1.78–4.69)	1.84 (1.02–3.32)	0.04*
	No	32 (32.0)	1.00	1.00		60 (37.3)	1.00	1.00	
Medical help-seeking behaviour	Yes	76 (76.0)	1.00	1.00		127 (78.9)	1.00	1.00	
	No	24 (24.0)	2.31 (1.08–4.94)	2.18 (1.01–4.70)	0.04*	34 (21.1)	2.09 (1.08–4.05)	1.98 (0.92–4.26)	0.08
Receipt of pain relief	Yes	68 (68.0)	3.02 (1.69–5.40)	1.92 (1.01–3.65)	0.04*	100 (62.1)	2.78 (1.72–4.49)	1.73 (0.96–3.12)	0.07
	No	32 (32.0)	1.00	1.00		61 (37.9)	1.00	1.00	
Preferred location for PC	At home	28 (28.0)	1.00	1.00		50 (31.1)	1.00	1.00	
	At nearby health centre	27 (27.0)	1.94 (0.92–4.09)	2.71 (1.12–6.56)	0.02*	40 (24.8)	1.85 (1.02–3.36)	2.58 (1.18–5.64)	0.01*
	At hospital	26 (26.0)	1.25 (0.60–2.60)	1.58 (0.68–3.67)	0.29	41 (25.5)	1.18 (0.66–2.11)	1.45 (0.68–3.09)	0.34
	At NGO facility	19 (19.0)	2.21 (0.98–4.98)	1.85 (0.72–4.75)	0.20	29 (18.0)	2.12 (1.05–4.28)	1.72 (0.73–4.05)	0.21
Social support level	Poor (3–8)	59 (59.0)	1.00	1.00		89 (55.3)	1.00	1.00	
	Moderate (9–11)	31 (31.0)	0.48 (0.25–0.92)	0.52 (0.25–1.08)	0.08	56 (34.8)	0.51 (0.31–0.84)	0.58 (0.32–1.05)	0.07
	Strong (12–14)	10 (10.0)	0.22 (0.09–0.54)	0.27 (0.10–0.73)	0.01*	14 (8.7)	0.25 (0.12–0.52)	0.31 (0.13–0.74)	0.01*
Cancer stage	Early (I–II)	42/92 (45.7)	1.00	1.00		76/175 (43.4)	1.00	1.00	
	Late (III–IV)	58/93 (62.4)	1.97 (1.09–3.56)	1.89 (1.01–3.54)	0.04*	85/120 (70.8)	3.17 (1.91–5.26)	2.84 (1.52–5.31)	0.001*
Receiving chemotherapy	Yes	92/123 (74.8)	4.82 (2.74–8.48)	4.21 (2.18–8.13)	<0.001*	123/212 (58.0)	1.64 (1.01–2.67)	1.58 (0.92–2.71)	0.09
	No	8/62 (12.9)	1.00	1.00		38/83 (45.8)	1.00	1.00	

PC, palliative care; COR, crude odds ratio; AOR, adjusted odds ratio; CI, confidence interval; NGO, non-governmental organization.

### Determinants among breast cancer patients

Among breast cancer patients (n = 295), 161 (54.6%) were classified as having higher PC needs. Muslim religious affiliation was significantly associated with higher PC needs among breast cancer patients (AOR = 2.18, 95% CI: 1.01–4.70, p = 0.04). Difficulty accessing the hospital was associated with 84% higher odds of unmet needs (AOR = 1.84, 95% CI: 1.02–3.32, p = 0.04). Preference for PC at a nearby health centre was associated with more than twofold higher odds of unmet needs compared with preference for home-based care (AOR = 2.58, 95% CI: 1.18–5.64, p = 0.01). Strong social support was highly protective, with breast cancer patients reporting strong support being 69% less likely to have higher PC needs (AOR = 0.31, 95% CI: 0.13–0.74, p = 0.01).

For breast cancer patients, not seeking medical help showed a trend toward higher needs but did not reach statistical significance in the adjusted model (AOR = 1.98, 95% CI: 0.92–4.26, p = 0.08). Similarly, receipt of pain relief showed a trend toward higher needs but was not statistically significant (AOR = 1.73, 95% CI: 0.96–3.12, p = 0.07).

Late-stage diagnosis was strongly associated with higher PC needs, with breast cancer patients diagnosed at late stages having nearly threefold higher odds of unmet needs compared with those diagnosed at early stages (AOR = 2.84, 95% CI: 1.52–5.31, p = 0.001). However, receiving chemotherapy among breast cancer patients was not significantly associated with higher PC needs in the adjusted model (AOR = 1.58, 95% CI: 0.92–2.71, p = 0.09). (**Table 8**).

**Table 8:** Caregiver burden and support characteristics by region among caregivers of women with breast and cervical cancer in Ethiopia, 2025 (N = 458).

Characteristic	Sidama (n = 59)	Addis ababa (n = 399)	Total (N = 458)	P-value	Odds ratio (95% CI) for Sidama *vs*. Addis Ababa
	n (%)	n (%)	N (%)		
Emotional strain (score ≥3)	49 (83.1)	297 (70.4)	346 (75.5)	0.003*	2.04 (1.01 – 4.15)
Financial burden (score ≥3)	39 (66.1)	235 (55.7)	274 (59.8)	0.008* ^8^	1.55 (0.87 – 2.76)
Preference for home-based care	25 (42.4)	124 (31.1)	149 (32.5)	0.08	1.63 (0.94 – 2.84)
Received palliative care training	5 (8.5)	43 (10.8)	48 (10.5)	0.59	0.77 (0.29 – 2.03)

Significant difference between regions at p < 0.05 (chi-square test)*.

Model fit statistics indicated good fit for both cancer-specific models (Cervical cancer: Hosmer-Lemeshow χ² = 5.92, df = 8, p = 0.66, pseudo-R² = 0.34; Breast cancer: Hosmer-Lemeshow χ² = 7.18, df = 8, p = 0.52, pseudo-R² = 0.29) (**Table 7**).

### Caregiver burden and support

Caregiver burden was assessed using the APCA Caregiver Burden Module. Among the 458 caregivers, the mean emotional strain score was 3.2 (SD = 1.1) on a 0–4 scale, with 346 caregivers (75.5%) reporting scores ≥3 indicating high emotional strain. The mean financial burden score was 2.9 (SD = 1.3), with 274 caregivers (59.8%) reporting scores ≥3 indicating high financial burden. Regional comparisons showed significantly higher burden among caregivers in Sidama compared with those in Addis Ababa (emotional strain: mean 3.6 *vs*. 3.1, p = 0.003; financial burden: mean 3.3 *vs*. 2.8, p = 0.008).

Preference for home-based care was reported by 149 caregivers (32.5%), with higher preference in Sidama (25 of 59 caregivers, 42.4%) than in Addis Ababa (124 of 399 caregivers, 31.1%). Access to PC training was low, with only 48 caregivers (10.5%) reporting any training, including 5 of 59 Sidama caregivers (8.5%) and 43 of 399 Addis Ababa caregivers (10.8%) (**Table 8**).

## Discussion

This study assessed palliative care needs among women with breast and cervical cancer attending two major Ethiopian referral hospitals. Using the mean SPICT-LIS score as the threshold for dichotomisation, 261 patients (54.4%, 95% CI: 49.8%–58.9%) were classified as having higher PC needs based on the composite score. This finding aligns with Ugandan study among patients with advanced cancer reported that 62% had significant unmet PC needs, particularly in pain management and psychosocial support (19). Similarly, research from Kenya among women with cervical cancer found that 58% reported inadequate symptom control and limited access to PC services (20). In Ethiopia, a rapid program evaluation in Addis Ababa and Sidama reported that female patients appreciated available PC services but found them insufficient to address their full range of needs (12). The consistency of these findings across the region underscores that unmet PC needs are not merely a local phenomenon but reflect systemic gaps in PC integration within oncology services across Sub-Saharan Africa.

The higher prevalence of psychosocial distress (72.1%) compared with physical symptoms (56.7%) challenges the traditional prioritization of physical symptom management in resource-limited PC programs. This finding suggests that in the Ethiopian context, the psychological and social consequences of a cancer diagnosis including stigma, fear, family disruption, and economic devastation may outweigh or compound physical suffering. This aligns with the WHO’s conceptualization of PC as requiring attention to physical, psychosocial, spiritual, and social dimensions of suffering (24).

The significantly higher psychosocial distress among breast cancer patients (75.6% *vs*. 66.5%, p = 0.03) likely reflects the unique psychosocial burden associated with breast cancer in the Ethiopian cultural context. Breast cancer affects a visible and culturally significant body part associated with femininity, motherhood, and sexuality. Mastectomy, a common treatment modality, can lead to body image disturbance, feelings of being incomplete as a woman, and fears about marital stability (2). Additionally, breast cancer is often associated with curse which may create additional psychological burden and stigma (3). The higher spiritual concerns among cervical cancer patients (55.1% *vs*. 51.5%), while not statistically significant, may reflect the stigma associated with a cancer perceived as related to sexually transmitted infection and promiscuity, leading to feelings of shame, guilt, and spiritual distress requiring religious or traditional healing interventions (4).

The similar overall magnitude of PC needs between breast and cervical cancer patients (54.6% *vs*. 54.1%, p = 0.91) despite differences in stage distribution (cervical cancer patients presented with more late-stage disease: 50.3% *vs*. 40.7%) suggests that breast cancer patients may experience unique needs that compensate for their earlier stage distribution. These may include body image concerns, fears about recurrence, and the psychological impact of treatment on femininity and marital relationships, as documented in other settings (9, 10).

The finding that Muslim religious affiliation was independently associated with higher PC needs among both cervical cancer (AOR = 2.35) and breast cancer patients (AOR = 2.18). This association remained significant after controlling for geographic location, access barriers, and sociodemographic factors. Several factors may contribute to this finding. First, there may be a lack of culturally competent PC services that align with Islamic practices related to palliative care as Islamic traditions emphasize specific practices around death and dying, including maintaining physical positioning and modesty (19). When healthcare providers are unfamiliar with or unable to accommodate these practices, patients may experience spiritual distress and avoid or delay engagement with formal PC services (20). Second, religious interpretations of suffering and fatalism the belief that illness and death are manifestations of divine will may influence perceptions of PC as unnecessary or as interfering with religious obligations (21). Similar findings have been reported in Tanzania, where Muslim communities demonstrated lower PC utilization related to religious beliefs about suffering (22). These findings underscore the need for culturally tailored PC approaches that engage religious leaders and community structures to improve access and acceptability.

Difficulty accessing the hospital was associated with significantly higher PC needs for both cervical cancer (AOR = 2.01) and breast cancer patients (AOR = 1.84). This finding aligns with research from Uganda, where geographic distance and transportation costs were identified as primary barriers to PC access, with patients traveling an average of 60 kilometres to reach the nearest PC provider (23). In Ethiopia, a study of cancer patients in Addis Ababa found that transportation costs and travel time were significant predictors of treatment interruption and poor symptom control (11).

The regional disparities observed throughout this study with Sidama patients experiencing significantly higher unmet PC needs (74.6% *vs*. 51.5%), greater access barriers (75.0% reporting transportation unaffordability *vs*. 60.6%), and more advanced disease at presentation (57.6% late-stage *vs*. 42.5%) reflect the broader urban-rural inequities in Ethiopian healthcare. Similar patterns have been documented in other low-income countries; a systematic review of PC in rural Sub-Saharan Africa found that patients in rural areas consistently had worse access, later diagnosis, and poorer outcomes compared with urban counterparts (21). These disparities highlight the limitations of Ethiopia’s centralized PC model, where specialized services are concentrated in the capital, leaving rural populations underserved (13). The finding supports calls for decentralized, community-based PC models that bring services closer to where patients live, reducing the access burden on patients and families (24).

Patients who reported not seeking medical help had significantly higher PC needs among cervical cancer patients (AOR = 2.18), with a similar trend among breast cancer patients (AOR = 1.98, p = 0.08). This identifies a population that remains outside the formal healthcare system until needs become severe. Avoidance of medical help-seeking may result from fear of cancer diagnosis, stigma associated with reproductive cancers, or fatalistic beliefs about treatment efficacy (25). Studies from Ethiopia have documented that stigma related to cervical cancer, including perceptions that it results from promiscuity or reflects poorly on a woman’s character, prevents many women from seeking timely care (3). Prior negative healthcare experiences, including long waiting times, perceived discrimination, or ineffective communication, may also discourage continued engagement with health services (26).

The finding that 65% of patients reported using traditional medicine, with higher rates in Sidama (74.6%), suggests that many patients seek help from informal sources rather than formal healthcare. This often represents a practical response to inaccessible or unaffordable formal care rather than simply cultural preference. Similar patterns have been observed in Malawi, where traditional healers serve as first-line providers for many cancer patients, and collaborative models between traditional and biomedical systems have shown promise in improving PC access (27). Integrating PC with traditional healing systems through mutual referral relationships and cross-training could potentially extend the reach of PC services to populations that currently avoid or cannot access formal healthcare.

The association between receipt of pain relief and higher PC needs among cervical cancer patients (AOR = 1.92), with a similar trend among breast cancer patients (AOR = 1.73, p = 0.07), reflects the relationship between symptom management and comprehensive PC. Patients receiving pain relief may continue to experience unmet needs in other domains even when pain is partially controlled (28) supported by the finding that 64.2% of patients receiving chemotherapy had higher PC needs compared with 31.7% of those not receiving chemotherapy, indicating that patients undergoing active treatment experience compounded needs. Additionally, in resource-limited settings where PC is often equated with pain management alone, patients may receive analgesics while psychosocial, spiritual, and economic needs remain unaddressed (29). Similar findings have been reported in Indian PC studies, where patients receiving pain medication continued to report high levels of distress related to family worries, financial concerns, and existential suffering (30). This suggests that in Ethiopia, pain management is often delivered in isolation rather than as part of integrated, comprehensive PC, highlighting the need for holistic assessment and multi-domain interventions that address the full spectrum of patient needs.

The association between preference for PC at a nearby health centre and higher unmet needs among both cervical cancer (AOR = 2.71) and breast cancer patients (AOR = 2.58) reflects the gap between current service delivery and patient preferences. Patients who express preference for local care are likely those who have experienced or anticipate difficulty accessing hospital-based services, as evidenced by the higher proportion of Sidama patients preferring home-based care (42.4% *vs*. 31.1% in Addis Ababa). This finding aligns with research from Kenya, where community-based PC models implemented through primary health centres significantly improved access and patient satisfaction compared with hospital-only models (31). In Ethiopia, the existing health extension program, with over 40, 000 health extension workers deployed at community level, represents an underutilized platform for PC integration (15). Studies have demonstrated that health extension workers can be trained to provide basic PC assessment, symptom monitoring, and psychosocial support, potentially addressing the access barriers identified in this study (32). The preference for local care also reflects cultural norms around dying at home and being cared for by family within familiar community settings, which is highly valued across Ethiopian cultures (33). Decentralizing PC services to primary health centres and community levels would align with both patient preferences and cultural values while addressing geographic access barriers.

Social support demonstrated a protective effect against higher PC needs for both cancer types. Among cervical cancer patients, strong social support was associated with 73% lower odds of higher PC needs (AOR = 0.27); among breast cancer patients, strong support was associated with 69% lower odds (AOR = 0.31). This finding is consistent with studies from Ethiopia examining social support in various health contexts. Research among people with mental illness in Gondar found that low social support was associated with poorer treatment outcomes and higher symptom burden (34). Similarly, a community-based study in Addis Ababa during the COVID-19 pandemic demonstrated that social support buffered against psychological distress and improved coping (35). Internationally, studies among cancer patients have consistently documented that social support improves quality of life, reduces psychological distress, and enhances treatment adherence (36, 37). The mechanisms through which social support exerts its protective effects likely include emotional reassurance that reduces anxiety and depression, practical assistance with activities of daily living and treatment navigation, financial support that alleviates economic hardship, and advocacy that helps patients access services (38). In the Ethiopian context, where extended family networks and community structures such as idir (traditional mutual aid associations) remain strong, leveraging these existing social resources may be particularly effective in reducing PC needs (16). Interventions that strengthen and mobilize community support networks could complement formal PC services and extend their reach, particularly in rural areas where formal services are limited.

Among cervical cancer patients, chemotherapy receipt was the strongest predictor of higher PC needs (AOR = 4.21), far exceeding the effect of late-stage diagnosis (AOR = 1.89). This suggests that the intensive treatment regimen for cervical cancer often involving concurrent chemoradiation with significant side effects including pain, nausea, fatigue, and gastrointestinal and genitourinary toxicity creates substantial palliative care needs independent of disease stage. Cervical cancer patients undergoing chemotherapy represent a particularly high-risk group requiring intensive palliative care support integrated with oncology care.

Among breast cancer patients, by contrast, late-stage diagnosis was the strongest predictor (AOR = 2.84), while chemotherapy receipt was not significantly associated with higher needs in the adjusted model (AOR = 1.58, p = 0.09). This may reflect differences in treatment protocols, with breast cancer chemotherapy often delivered in outpatient settings with different symptom profiles, or may indicate that breast cancer patients’ needs are more strongly driven by stage-related factors such as metastatic disease burden than by treatment modality. Breast cancer patients with late-stage disease should be prioritized for comprehensive palliative care assessment regardless of treatment modality.

The finding that 75.5% of caregivers reported high emotional strain and 59.8% reported high financial burden is consistent with studies from other Sub-Saharan African settings. Research from Uganda found that family caregivers of cancer patients experienced significant psychological distress, with 70% meeting criteria for anxiety or depression, and reported substantial economic impacts including loss of income and depletion of savings (39). In Tanzania, a study of caregivers for women with cervical cancer documented that caregiving responsibilities interfered with employment and resulted in food insecurity for the entire household (40).

The regional differences observed, with Sidama caregivers reporting significantly higher burden across all measures, likely reflect the compounded challenges of caring for patients with more advanced disease (57.6% late-stage in Sidama *vs*. 42.5% in Addis Ababa) while having less access to formal support services, including the near-absence of PC training (8.5% in Sidama *vs*. 10.8% in Addis Ababa). The low rates of caregiver training across both settings (overall 10.5%) represent a critical gap, as caregiver education and support have been shown to reduce burden and improve patient outcomes in PC contexts (17). Given that Ethiopian families operate within a collectivist cultural framework where extended family members assume primary responsibility for patient care, often with minimal formal support (16), interventions that provide caregiver training, respite care, and financial support could significantly reduce burden and improve the quality of care patients receive.

### Strength and limitation of the study

This study’s strengths include its large sample, use of validated instruments adapted for the Ethiopian context, and inclusion of both patients and caregivers across two diverse regions. The multidimensional assessment across physical, psychosocial, spiritual, and economic domains provides a comprehensive understanding of PC needs. The cancer-type specific analyses and examination of regional variations allow for targeted recommendations. However, phone-based recruitment may have excluded marginalized populations without telephone access. Reliance on self-reported data introduces potential recall and social desirability bias. Finally, the study was conducted at only two referral hospitals, limiting generalization to those not accessing formal healthcare.

## Conclusion, and recommendation

This study was conducted to understanding the multifaceted palliative care needs of women with breast and cervical cancer and their caregivers in Ethiopia, where cancer burdens are rising but palliative services remain severely limited. The findings reveal that psychosocial distress surpasses physical symptoms as the most prevalent unmet need, particularly among breast cancer patients, while cervical cancer patients face compounded challenges related to chemotherapy. Significant regional disparities exist, with patients in Sidama experiencing higher unmet needs than those in Addis Ababa, driven by geographic access barriers, advanced disease at presentation, and limited service availability. Strong social support emerged as a protective factor against higher palliative care needs, and patients preferred receiving care at local health centres, highlighting the misalignment between current centralized services and patient preferences. Caregivers bear an enormous burden, with three-quarters reporting high emotional strain and most experiencing financial hardship, yet none receive any formal training or support. These findings implies the need for policy action: Ethiopia have to prioritize decentralizing palliative care through integration into primary health systems, leveraging the existing health extension program to bring services closer to communities. Cancer treatment protocols should embed palliative care from diagnosis onward referring cervical cancer patients receiving chemotherapy and ensuring breast cancer patients receive psychosocial support addressing body image and marital concerns. Culturally competent care models have to be developed in partnership with religious, and community leaders. Finally, comprehensive caregiver support programs encompassing training, respite care, and economic assistance are essential to sustain the family-based caregiving that underpins Ethiopia’s collectivist cultural framework. Without such systemic reforms, the palliative care needs of Ethiopian women with cancer and their caregivers will remain significantly high.

## Data Availability

The raw data supporting the conclusions of this article will be made available by the authors, without undue reservation.
